# Development of Healthy, Nutritious Bakery Products by Incorporation of Quinoa

**DOI:** 10.3390/foods8090379

**Published:** 2019-09-01

**Authors:** Jaime Ballester-Sánchez, M. Carmen Millán-Linares, M. Teresa Fernández-Espinar, Claudia Monika Haros

**Affiliations:** 1 Institute of Agrochemistry and Food Technology (IATA-CSIC), 46980 Valencia, Spain; 2 Vegetable Protein Group, Instituto de la Grasa (IG-CSIC), 41013 Seville, Spain

**Keywords:** *Chenopodium quinoa*, bakery products, DRIs/DRVs (Dietary Reference Intakes/Dietary Reference Values) and AI (Adequate Intake), FAO (Food and Agriculture Organization), EFSA (European Food Safety Authority), protein quality, polyunsaturated fatty acids, dietary fibre, mineral availability, glycaemic index estimation

## Abstract

The use of quinoa could be a strategy for the nutritional improvement of bakery products. The inclusion of this pseudocereal, with its suitable balance of carbohydrates, proteins, lipids and minerals, could contribute to attaining the adequate intake values proposed by the FAO (Food and Agriculture Organization) and/or EFSA (European Food Safety Authority) for suitable maintenance and improvement of the population’s health. Bakery products made with white, red or black royal quinoa significantly improved the contribution to an adequate intake of polyunsaturated fatty acids (linoleic and linolenic acids) and dietary fibre, which produced an improvement in the soluble/insoluble fibre ratio. There was also an increase in the contribution to the average requirement of Fe and Zn, although the increase in the phytate/mineral ratio would make absorption of them more difficult. Inclusion of flour obtained from the three quinoas studied slightly improved the protein quality of the products that were prepared and positively affected the reduction in their glycaemic index.

## 1. Introduction

Quinoa is a native pseudocereal of Latin America that now has great consumer acceptance in Europe and throughout the world. Because of its suitable balance of carbohydrates, proteins, lipids and minerals and its bioactive compound content, it has been proposed that it should be included as a strategy to improve the nutritional quality of bakery products made with refined flours [1,2]. Not only would the incorporation of quinoa flour in formulations increase the protein content but it could also improve the biological value of the proteins in these formulations, since quinoa proteins contribute essential amino acids that are limiting in wheat flours (such as lysine and threonine), and they are more digestible [3]. It could also lead to an increase in the unsaturated fatty acid content and an improvement in the omega 3/omega 6 fatty acid relationship. The main unsaturated fatty acids in quinoa are linoleic and α-linolenic acids, a precursor of long-chain polyunsaturated fatty acids (PUFAs), which are essential fatty acids [1,4]. Moreover, its high fibre and mineral contents could help to attain the daily requirements of these substances and of calcium, iron and zinc in the diet [1]. However, mineral bioavailability does not depend only on the concentration of the mineral in question in the food (such as Ca, Fe or Zn); there are compounds such as phytates that form complexes with di- and trivalent minerals and prevent their absorption [5]. Because of the high proportion of dietary fibre in wholemeal flours made from quinoa and other grains, their inclusion in bread formulations could have a beneficial effect by improving gastrointestinal transit and reducing levels of cholesterol and as a source of prebiotics, among other functions of dietary fibre [6]. On the other hand, there are studies that propose strategies to reduce the glycaemic response in bakery products by the use of whole grains and by incorporating the external parts of the grain [7]. Diets with a high glycaemic index (GI) are associated with the development of metabolic dysfunction and predisposition to type 2 diabetes, as well as with problems of overweight/obesity [8].

The dietary reference intakes (DRIs) proposed by the FAO/WHO (Food and Agriculture Organization/World Health Organization) for the world population, also known as dietary reference values (DRVs) proposed by EFSA (European Food Safety Authority) for the European population, are a set of nutrient reference values that indicate the quantity of a nutrient that must be consumed regularly to maintain the health of a healthy person (or population). The main aim of the first reference values, proposed in the early 1940s, was to prevent nutritional deficiencies in the population [9]. However, nutrient reference intake values also focus on preventing illness and on promoting health [10]. There may be differences in the reference values proposed by the two organisations because they are based on the average intakes of the population in question, taking their food behaviour into account. These reference values have become a fundamental tool for evaluating the nutrients provided by a food when it is ingested.

The aim of this study was to make a detailed analysis of the chemical composition of the raw materials and the products developed, including the nutritional profile of products in which wheat flour was replaced with whole white, red or black quinoa flour. The contribution made to the DRIs/DRVs of nutrients such as linoleic acid, linolenic acid, calcium, iron, zinc and dietary fibre by the ingestion of a bakery product with quinoa was investigated and the impact on the glycaemic index was estimated.

## 2. Materials and Methods

### 2.1. Materials

White, red and black quinoa seeds (organic “*quinoa real*”, royal quinoa), marketed by ANAPQUI (La Paz, Bolivia), were purchased from Ekologiloak (Bizkaia, Spain). The three types of quinoa seeds were ground separately to obtain the corresponding flour by using a commercial blender three times for 30 s at room temperature (Aromatic, Taurus, Oliana, Spain) and were stored at 14 °C. Dehydrated yeast (*Saccharomyces cerevisiae*, Maizena, Spain) was used as a starter and commercial wheat flour from a local supermarket (Carrefour, Spain) was used for the breadmaking process.

### 2.2. Breadmaking Procedure

The control bread dough formula consisted of wheat flour (500 g), dehydrated yeast (1.0 g/100 g flour), sodium chloride (1.6 g/100 g flour) and distilled water (70.8 g/100 g flour). Whole quinoa flour was incorporated in the bread dough formula at a proportion of 25 g/100 g flour. The breadmaking procedure was performed as described in a previous paper [11]. Measurements were carried out in triplicate.

### 2.3. Proximate Chemical Composition

Proximate analysis of moisture, dietary fibre, starch and phytic acid (*myo*-inositol 1,2,3,4,5,6-hexakisphosphate) of the raw materials and breads was performed according to approved methods 925.09, 991.43, 996.11 and 986.11, respectively [12]. Protein determination was carried out by the Dumas combustion method (N conversion factor 5.7) according to ISO/TS 16634-2 [13]. Lipid and ash contents were determined according to Official Methods 30-10 and 08-03, respectively [14]. Measurements were carried out in triplicate.

### 2.4. Amino Acid Analysis

Samples (1 g) were hydrolysed with 4 mL of 6 N HCl. The solutions were sealed in tubes under nitrogen and incubated in an oven at 110 °C for 24 h. Amino acids were determined in the acid hydrolysis, after derivatisation with diethyl ethoxymethylenemalonate, by high-performance liquid chromatography (HPLC) Model 600E multi-system with a 484 UV–Vis detector (Waters, Milford, MA, USA) with a 300 mm × 3.9 mm reversed-phase column (Novapack C18, 4m; Waters) at 18 °C); acetonitrile in binary gradient, the detection at 280 nm, with D-L aminobutyric as standard, Sigma Chermical Co. St. Louis, MO, USA) according to the method of Alaiz et al. [15]. The amino acid composition was expressed as grams of amino acid per 100 g of protein. Measurements were carried out in triplicate.

### 2.5. Essential Amino Acid Index

The essential amino acid index (EAAI) was calculated according to Motta et al. [16], applying the following equation:

*EAAI* = 10*^log^*^*EAA*^ (1)

where

(2) $$EAA=0.1\left[\log\left(\frac{a_{1}}{a_{1s}}\times 100\right)+\log\left(\frac{a_{2}}{a_{2s}}\times 100\right)+\ldots \log\left(\frac{a_{n}}{a_{ns}}\times 100\right)\right]$$

$a_{1}$, …, $a_{n}$ are the amino acid contents in the sample, and $a_{1s}$, …, $a_{ns}$ are the essential amino acid requirements in the protein standard [17].

### 2.6. Fatty Acid Composition

The samples were transesterified to convert fatty acid methyl esters (FAMEs), following the methodology previously described by Garces and Mancha [18]. The fatty acid composition and quantification were determined using an Agilent Technologies chromatograph (Santa Clara, United States) with a capillary column (100 m × 0.25 mm i.d. (internal diameter) × 0.25 µm film thickness) and a flame ionisation detector according to IUPAC (International Union of Pure and Applied Chemistry) Method 2.302 [19]. Measurements were carried out in triplicate.

### 2.7. Mineral Composition

The total Ca, Fe and Zn concentrations in samples were determined using a flame absorption spectrometer at the Analysis of Soils, Plants and Water Service at the Institute of Agricultural Sciences, Madrid, Spain. Each sample (0.5 g) was placed in a Teflon perfluoroalkoxy vessel and digested by means of HNO_3_ (4 mL, 14 M) and H_2_O_2_ (1 mL, 30% *v/v*) attack. Samples were irradiated at 800 W (15 min at 180 °C) in a Microwave Accelerated Reaction System (MARS, Charlotte, NC, USA). At the end of the digestion programme, the digest was placed in a polypropylene tube and made up to final volume with 5% HCl. Measurements were carried out in triplicate.

### 2.8. In Vitro Digestion and Glycaemic Index (GI)

In vitro starch digestion and glycaemic index estimation were performed according to the modified method reported by Sanz-Penella et al. [7]. The hydrolysis index (HI) was calculated from the area under the curve (AUC) from 0 to 120 min for samples as a percentage of the corresponding area of reference (wheat bread) (HI = AUC_sample_/AUC_wheat bread_ × 100). The glycaemic index (GI) was calculated with the equation GI = 0.549 × HI + 39.71 used by Laparra et al. [20] for the inclusion of Latin American crops in bread. The measurements were carried out in triplicate. The predicted glycaemic load (pGL) was calculated for a 100 g bread portion from the glucose-related GI according to pGL= glycaemic index × total carbohydrates/100, taking into account the total carbohydrates of each sample [21].

### 2.9. Statistical Analysis

One-way ANOVA and Fisher’s least significant differences (LSD) were applied to establish significant differences between samples. All statistical analyses were carried out with the Statgraphics Plus 16.1.03 software (Bitstream, Cambridge, MN, USA), and differences were considered significant at *p* < 0.05.

## 3. Results and Discussion

### 3.1. Raw Material and Bread Chemical Composition

The protein contents of the raw materials did not reveal significant differences between the quinoa flours or with the control flour (Table 1). The control flour had a slightly lower protein content in comparison with other coloured quinoa varieties reported in the literature [22]. These differences could be due mainly to the use of a lower protein conversion factor (N × 5.7) than the one generally used in the literature (N × 6.25). The quinoa nitrogen-to-protein conversion factor used in the literature varies from 5.70 to 6.25 [1]. In this regard, the European Union proposes setting a single value, but so far, the value has not been agreed [23].

In the bread formulations, replacing 25% of the wheat flour with whole quinoa flour produced a significant increase in the protein content in breads made with red and black quinoa in comparison with the control sample (Table 1). The lipid contents in the quinoa flours were significantly higher than those of the wheat flour, basically because the germ had been removed from the wheat flour (Table 2). However, it must be pointed out that the lipid contents of quinoa grains are higher than those of whole wheat flour mainly because of the greater proportion of the germ in relation to the other anatomical parts of the quinoa grain [1]. In the comparison of varieties, the lipid contents were significantly higher in the white and red quinoas than in the black variety (Table 2).

The results are in agreement with the values found in the literature, which range between 4.1 and 9.7 g/100 g, indicating great variability between quinoa varieties [1]. The predominance of unsaturated fatty acids in quinoa seeds is well known [1], and therefore, the greater lipid content in bakery products made with quinoa could help to improve the saturated/unsaturated fatty acids ratio in Western diets [24].

The ash analysis did not reveal significant differences (*p* < 0.05) between the various quinoa flours or between the products made with them (Table 3). The same tendency was observed with the total dietary fibre (TDF) contents of the quinoa flours, which were significantly higher than those of the wheat flour (Table 4). Although the comparison in this study was made with refined wheat flour, it has been reported in the literature that quinoa grain generally has a higher total dietary fibre content than wheat grain [1]. Even though there were no significant differences between white and red quinoa with regard to the dietary fibre content (SDF, soluble dietary fibre; IDF, insoluble dietary fibre; and TDF), an increasing tendency was observed in the quinoa flours: white<red<black (Table 4). Diaz-Valencia et al. [22] reported similar behaviour with regard to the total fibre content of coloured quinoas from Peru. The dietary fibre content of the bakery products showed a content that was related to the contents of the various raw materials, with black quinoa bread having the highest total dietary fibre content (Table 4). Soluble/insoluble relationships close to 1:2 have demonstrated a more effective physiological action [25]. The bakery products that showed a ratio close to the recommended value were the ones made with red and white quinoa (Table 4). The improvement in the ratio in the bakery products could have a hypocholesterolaemic effect, attributable to the higher SDF content, as reported by Konishi et al. [26] in their study of the effect of quinoa seed pericarp on mice. It has also been reported that SDF reduces gastric emptying, the glucose absorption rate and postprandial insulin, and therefore, an increase in the content of this fibre in bakery products could help to improve the control of glycaemia in blood [27]. With regard to the intake of total dietary fibre, both the FAO and EFSA propose an adequate intake (AI) of 25 grams per day for adults [28,29].

Mean consumption of 100 g of bread made with quinoa flour helped to attain 34%–43% of the daily adequate intake of fibre in adults, and black quinoa bread was the one that produced the highest percentage contribution, increasing the contribution by 19% in comparison with the control sample (Table 4).

The starch values of the quinoa flours were significantly lower than that of the wheat flour [11]. However, whole flours have a lower percentage of starch than refined flours, and the quinoa grains used in this study had even lower starch contents than the levels reported for flours of whole cereals such as wheat, barley and corn [1]. The starch content of the white variety was significantly higher than that of the red and black varieties [11]. Similar results were found in the literature for white quinoa grown in Holland or in Peru [22,31]. With regard to the starch contents of the bakery products, no significant differences were observed, but there was a similar tendency to the one seen in the analysis of the raw materials, with the starch content in the breads made with quinoa decreasing to a level of 25% (Table 5). This reduction could lead to a decrease in the glycaemic load of products made with quinoa flour, as described below [21].

### 3.2. Amino Acid Composition

The amino acid contents of the raw materials and the breads are shown in Table 1. The predominant amino acid in the quinoa flours was glutamic acid, and it was significantly lower than in the wheat flour. Similar results have been found in cultivars in various regions [1,16]. The sulfur-containing amino acids (methionine and cysteine) had the lowest levels in the flours analysed, as also reported by other researchers [16]. In general, the essential amino acid contents in quinoa were higher than those reported in most whole cereal grains, such as wheat, barley, rice and/or corn [3]. Consequently, one would expect that the incorporation of 25% of these flours in bread formulations would produce a significant increase (*p* ˂ 0.05) in the essential amino acid contents. In fact, the concentrations of histidine, threonine and lysine increased significantly in comparison with the control sample.

However, although the incorporation of 25% of quinoa flour produced a general improvement in the amino acid profile of the bakery products developed, the improvement did not reach the value suggested by the FAO [32] for lysine (4.5 g/100 g of protein). The improvement in the nutritional quality of the protein provided by the quinoa raw materials and the bakery products prepared with quinoa was evaluated by calculating the EAAI (Table 1). The EAAI values of the quinoa flours were slightly higher than that of the wheat flour, indicating a protein of greater nutritional quality (Table 1). However, the incorporation of 25% of quinoa flour in the breads produced almost no change in the EAAI values in comparison with the control sample. Apparently, there were also no losses during baking, taking the lysine values of the raw materials as a reference for the theoretical calculation (data not reported). An increase in the percentage of quinoa flour (over 50%) could attain the lysine values proposed by the FAO [32].

### 3.3. Fatty Acid Composition

The analysis of the fatty acid profile of the raw materials showed higher levels in the quinoa flours than in the control flour (Table 2). A noteworthy result was the significantly lower concentrations of palmitic, stearic and oleic acids in the black quinoa flour in comparison with the other quinoas, mainly due to the lower lipid contents of the flour of this variety. However, there were no significant differences in the essential fatty acid contents in the various varieties of quinoa; linoleic acid was the main fatty acid (over 50%), followed by oleic acid (over 20%), as reported in the literature [1]. The higher concentrations of monounsaturated fatty acids (MUFAs) and polyunsaturated fatty acids (PUFAs) in the quinoa flours in comparison with the wheat flour produced a significant change (*p* ˂ 0.05) in the lipid profile of the bakery products developed. Accordingly, intake of these products could help to reduce the risk of suffering certain diseases. It has been reported that replacing saturated fatty acids (SFAs) with polyunsaturated fatty acids (PUFAs) and/or monounsaturated fatty acids (MUFAs) in intake helps to reduce the LDL cholesterol concentration and the total cholesterol/HDL cholesterol ratio, and therefore the risk of suffering heart disease [32]. Consequently, adequate intakes of 2.5 E% (percentage of energy intake) per day [32] or 4 E% per day [29] of the energy intake have been proposed for linoleic acid (LA), and 0.5 E% per day for linolenic acid (ALA) [29,32]. The lipid profile analysis showed a significant increase in LA and ALA in the breads with quinoa, which generated an increase in the contribution to AIs (Table 2).

Consumption of 100 g of bread with quinoa would contribute up to 15 E% (according to the FAO) or up to 9 E% (according to EFSA) of LA and up to 8 E% of ALA [29,32]. The saturated/unsaturated acid ratio is an indicator for nutritional and functional analysis [24]. In the present study, the saturated/unsaturated fatty acids ratio of the products made with quinoa was higher than that of the control bread, mainly because of their high linoleic and oleic acid contents, which could help to reduce the incidence of cardiovascular diseases [33]. Omega 6 (n-6) and omega 3 (n-3) fatty acids are essential for humans and can only be biosynthesised from their ALA and LA precursors [1]. There is no scientific rationale for recommending a specific n-6 to n-3 ratio, or LA to ALA ratio, if intakes of n-6 and n-3 fatty acids lie within the recommendations established or previously reported [28,29]. However, in order to facilitate labelling, adequate intake values of 10 g of LA/day and 2 g of ALA/day have been proposed for adults, from which it is possible to establish a ratio of 5:1 [34]. The current n-6/n-3 ratio in Western diets has been estimated as lying in the range of 14:1–20:1 [33]. Accordingly, intake of the products developed in the present study could help to improve the imbalance in Western diets and thus help to achieve the recommendations of the international organisations.

### 3.4. Mineral and Phytate Composition

There were no significant differences in the Fe contents of the quinoa flour, whereas the Ca content was significantly higher in the black quinoa, followed by the white and red quinoas (Table 3). The red quinoa had the highest Zn content, followed by the black and white quinoas. Studies on other pseudocereals found higher Ca and Mg contents in coloured genotypes [35]. However, Diaz-Valencia et al. [22] did not find a colour effect in their study with various varieties of quinoa. The variability of the mineral contents in the grains can be explained by the agroecological conditions in which they are grown, especially the soil [3]. The values reported in the present work are of the same order as those reported for other quinoas and, in general, for other whole grains [1]. It is known that minerals in wheat and other cereals are located mostly in the outer parts of the grain [36]. Accordingly, as was to be expected, the quinoa flours had significantly higher mineral contents than the control, which caused the same tendency in the breads made with those flours. The analysis of the bakery products with quinoa showed a significant increase in mineral contents with the exception of Ca, which was only significant in the product made with black quinoa (*p* ˂ 0.05). The increase in the Fe and Zn contents in the products made with quinoa could have a significant impact on the consumer, helping to attain the DRIs/DRVs proposed by the FAO/EFSA, respectively [29,30]. The contribution of minerals to the diet made by the bakery products is shown in Table 3. Ingestion of 100 g of bread made with quinoa did not improve the contribution to the average requirement (AR) for Ca, but it increased the contribution to the AR of Fe by 4%–5% (AR_FAO_: 14 mg/day; AR_EFSA_: 11/16 mg/day) in comparison with the white bread. With regard to Zn, various studies have demonstrated the negative effect of phytic acid on the bioavailability of this mineral and other di- and trivalent minerals [1]. Phytates are negatively charged at physiological pH and can therefore form insoluble complexes with cations in the digestive tract, thus reducing their bioavailability [5]. Because of this inhibitory effect, particularly for Zn, the FAO and EFSA have both proposed various ARs for the consumption of phytates in the diet. The FAO [30] considers three levels of bioavailability of zinc, depending on the phytate content in the diet (high, moderate and low bioavailability), whereas EFSA [29] contemplates four levels of phytate intake per day (300, 600, 900 and 1200 mg per day). The incorporation of 25% of whole quinoa flour in bakery products generated an increase of ~13%/17% (FAO) and ~6%/7% (EFSA) in the contribution to the AR of Zn for males/females, respectively, in comparison with the control sample in diets with high bioavailability (phytate/zinc ˂ 5). In diets with high consumption of phytates or low bioavailability (phytate/zinc ≥15) the contribution increased by only ~4%/5% (FAO) and ~3%/4% (EFSA) for males/females, respectively.

The significantly higher (*p* < 0.05) phytic acid content of the quinoa flours with respect to the wheat flour caused an increase in the phytic acid content in the breads made with quinoa (Table 3). The reduction in the phytate content in the products made in comparison with the raw materials was basically due to the activity of phytases, which are activated during the kneading and fermentation stages and the first stages of baking, causing hydrolysis of the phytates to *myo*-inositol with a lesser degree of phosphorylation [37]. Phytate/mineral molar ratios are a useful tool for predicting the inhibitory effect on the bioavailability of minerals in humans [38]. Phytate/Ca ratios greater than 0.24 in a food indicate that after ingestion the bioavailability of that mineral could be compromised. In the case of Fe, the bioavailability is compromised if the phytate/Fe ratio is greater than 1. Similarly, absorption of Zn is drastically reduced when the phytate/Zn ratio is greater than 15 [38]. The breads made with 25% of quinoa flour had phytate/Fe ratios greater than 1 (2.9–3.70), which would negatively affect absorption of this mineral. However, the phytate/Ca ratios in the products with quinoa were less than 0.24, and the phytate/Zn ratios were less than 15 in the formulations with inclusion of quinoa flour, thus improving the bioavailability of these two minerals, mainly because of the greater contribution of these minerals, despite the higher concentration of phytates in the quinoa flour (Table 3).

### 3.5. Glycaemic Index

The analyses performed for the glycaemic index estimation are shown in Table 5. After 90 min of digestion of the wheat bread it showed 82% of hydrolysed starch. The TSH_90_ (total starch hydrolysed at 90 min) was reduced by 9%–11% in the breads with quinoa. The wheat bread showed the significantly (*p* < 0.05) highest glycaemic index (GI) percentage in comparison with the breads made with whole quinoa flour (Table 5). Furthermore, significantly higher values were observed in the GI of the breads made with black quinoa compared with those made with white and red quinoa. In the literature, a reduction of ~5% was reported in the GI of bread made with 100% of quinoa in comparison with the reference control bread [21]. With regard to the predicted glycaemic load (pGL), significant differences were observed between all the bread samples analysed (Table 5), with the bread made with black quinoa being the sample that showed the smallest pGL. The Lineweaver–Burk plot, widely accepted and established for calculating the kinetic parameters of starch hydrolysis, was used to transform cumulative curves into linear curves [7]. With this method it is possible to calculate the reciprocal values of (% of starch hydrolysis) and time. The inclusion of 25% of quinoa did not produce significant changes in the hydrolysis coefficients (Table 5). However, the values of the slope of the curve in the Lineweaver–Burk plot showed a smaller slope, indicating faster hydrolysis of starch, in the wheat bread in comparison with the breads with quinoa. This may have been because the fibre and other compounds present in quinoa, such as polyphenols, affected the glucose uptake kinetics, as reported by other researchers [7,39].

Moreover, in vivo studies have indicated that breads formulated with different sources of dietary fibre or mixtures of them in baked products have a hypoglycaemic effect in humans owing to the reduction in the rate of absorption of carbohydrates from the diet because of the formation of a viscous gel in the small intestine [40]. In this context it must be emphasised that the glycaemic response of foods depends on the texture and size of the particles, but also on the type of starch, the degree of its gelatinisation, the type of association/interaction with other components of the food, and the type of processing of the food. Therefore, the differences found in the products with quinoa were due not only to an effect of dilution of starch as a result of the inclusion of a whole flour but also to the different properties of quinoa starch, among other factors.

## 4. Conclusions

Replacement of 25% of wheat flour with white, red or black quinoa flour produced a general improvement in the nutritional profile of the bakery products developed in this study in terms of an improvement in the contribution to adequate intake of fibre, general increase in protein content with a slight improvement in the amino acid profile, especially in lysine, and an increase in lipid content with an improvement in the saturated/unsaturated fatty acids ratio due to the higher content of linoleic acid in the quinoa flours, helping to attain adequate intake of linoleic and linolenic acids. The mineral content of the quinoa flours produced an improvement in the contribution to the average requirements of Fe and Zn made by the breads with addition of quinoa, although an increase in the phytate/mineral ratio might compromise absorption of these minerals. The breads with quinoa flour also produced a reduction in the glycaemic index and the predicted glycaemic load, with a tendency for the starch hydrolysis rate to decrease.

## Figures and Tables

**Table 1:** Amino acid composition of raw materials and breads.

Amino Acid g/100 g Prot	Target	Flours					Breads		
	FAO ^#^	Control	White Quinoa	Red Quinoa	Black Quinoa	Control	White Quinoa	Red Quinoa	Black Quinoa
Proteins, % d.m. ^a^		13.3 ± 0.1 abc	13.0 ± 0.7 abc	12.8 ± 0.7 ab	13.5 ± 0.2 abc	12.5 ± 0.3 a	13.2 ± 2.5 abc	14.7 ± 0.2 c	14.4 ± 0.2 bc
Asp		4.5 ± 0.1 a	10.5 ± 0.2 c	10.44 ± 0.04 c	10.6 ± 0.1 c	4.4 ± 0.3 a	6.0 ± 0.2 b	6.0 ± 0.1 b	5.8 ± 0.1 b
Glx		36.9 ± 1.9 c	16.7 ± 0.4 a	16.6 ± 0.2 a	16.51 ± 0.06 a	37.5 ± 1.1 c	31.9 ± 1.2 b	32.3 ± 0.7 b	31.9 ± 0.8 b
Ser		5.3 ± 0.3 a	5.2 ± 0.1 a	5.17 ± 0.05 a	5.22 ± 0.04 a	5.3 ± 0.5 a	5.4 ± 0.1 a	5.41 ± 0.07 a	5.3 ± 0.1 a
Gly		3.8 ± 0.2 a	5.8 ± 0.1 c	5.56 ± 0.04 c	5.80 ± 0.07 c	3.8 ± 0.4 a	4.3 ± 0.1 b	4.4 ± 0.1 b	4.3 ± 0.1 b
Arg		3.6 ± 0.2 a	9.1 ± 0.3 c	9.1 ± 0.2 c	8.7 ± 0.1 c	3.4 ± 0.4 a	4.4 ± 0.1 b	4.62 ± 0.07 b	4.3 ± 0.1 b
Ala		3.0 ± 0.1 a	5.0 ± 0.1 c	4.95 ± 0.01 c	5.0 ± 0.1 c	3.2 ± 0.3 a	3.7 ± 0.1 b	3.8 ± 0.1 b	3.6 ± 0.1 b
Pro		14.5 ± 2.2 e	6.66 ± 0.06 a	8.3 ± 0.3 ab	8.37 ± 0.03 abc	10.5 ± 0.4 cd	10.2 ± 0.6 bcd	8.9 ± 0.8 bcd	10.8 ± 0.3 d
	**Essential amino acids (EAA)**								
His	1.5	3.00 ± 0.07 c	3.81 ± 0.05 d	3.80 ± 0.01 d	3.91 ± 0.08 d	2.3 ± 0.2 a	2.61 ± 0.08 b	2.58 ± 0.04 b	2.66 ± 0.05 b
Val	3.9	3.9 ± 0.1 a	4.9 ± 0.1 c	4.85 ± 0.04 c	5.0 ± 0.1 c	4.1 ± 0.2 ab	4.2 ± 0.2 b	4.3 ± 0.1 b	4.2 ± 0.1 ab
Met	1.6	0.6 ± 0.3 a	0.6 ± 0.4 a	0.7 ± 0.2 a	0.71 ± 0.07 a	0.9 ± 0.1 a	0.7 ± 0.1 a	0.87 ± 0.01 a	0.7 ± 0.1 a
Cys	0.6	1.7 ± 0.1 d	1.0 ± 0.1 ab	1.06 ± 0.05 ab	1.0 ± 0.2 a	1.4 ± 0.3 cd	1.23 ± 0.04 abc	1.37 ± 0.08 c	1.3 ± 0.1 bc
Ile	3.0	3.5 ± 0.1 a	4.2 ± 0.1 c	4.23 ± 0.08 c	4.19 ± 0.06 c	3.7 ± 0.2 ab	3.7 ± 0.1 ab	3.77 ± 0.07 b	3.7 ± 0.1 ab
Leu	5.9	6.8 ± 0.3 a	7.2 ± 0.1 a	7.27 ± 0.07 a	7.24 ± 0.09 a	6.8 ± 0.7 a	7.0 ± 0.3 a	7.1 ± 0.1 a	6.9 ± 0.1 a
Phe	3.8 *	4.7 ± 0.2 ab	4.3 ± 0.1 a	4.34 ± 0.05 a	4.28 ± 0.03 a	4.6 ± 0.5 ab	4.6 ± 0.1 ab	4.7 ± 0.1 b	4.6 ± 0.1 ab
Tyr		2.7 ± 0.4 bcd	3.0 ± 0.1 d	2.9 ± 0.1 d	2.9 ± 0.1 cd	2.49 ± 0.06 abc	2.1 ± 0.1 a	2.4 ± 0.1 ab	2.2 ± 0.2 a
Lys	4.5	1.8 ± 0.1 a	6.2 ± 0.1 c	6.05 ± 0.06 c	6.21 ± 0.07 c	2.0 ± 0.2 a	2.8 ± 0.2 b	2.76 ± 0.01 b	2.7 ± 0.1 b
Thr	2.3	2.8 ± 0.1 a	4.31 ± 0.08 c	4.19 ± 0.06 c	4.29 ± 0.03 c	2.9 ± 0.3 a	3.3 ± 0.1 b	3.31 ± 0.05 b	3.24 ± 0.09 b
EAAI		2.66	2.84	2.85	2.86	2.68	2.69	2.73	2.69

Values are expressed as mean ± standard deviation (*n* = 3). Values followed by the same letter in the same line are not significantly different at 95% confidence level. ^a^ Dry matter. ^#^ Amino acid pattern suggested by FAO for adults (g/100 g protein). * Suggested composition for aromatic amino acids Phe + Tyr (FAO, 2008). Prot.: proteins; d.m.; dry matter; Asp, Aspartic Acid; Glx, Glutamate or Glutamine; Ser, Serine; Gly, Glycine; Arg, Arginine; Ala, Alanine; Pro, Proline; His, Histidine; Val, Valine; Met, Methionine; Cys, Cysteine; Ile, Isoleucine; Leu, Leucine; Phe, Phenylalanine; Tyr, Tyrosine; Lys, Lysine; Thr, Threonine; FAO, Food and Agriculture Organization; EAAI, essential amino acid index.

**Table 2:** Fatty acid composition of raw materials and breads and adequate intake contributions.

**Organic Acid**		**Units ^a^**	**Flours**			
			**Control**	**White Quinoa**	**Red Quinoa**	**Black Quinoa**
Lipids		g/100 g	1.2 ± 0.1 a	5.4 ± 0.6 c	5.3 ± 0.2 c	4.5 ± 0.4 b
Palmitic acid	C16:0	mg/g	0.33 ± 0.03 a	0.56 ± 0.07 c	0.57 ± 0.03 c	0.45 ± 0.05 b
Stearic acid	C18:0	mg/g	0.02 ± 0.01 a	0.05 ± 0.01 c	0.05 ± 0.01 c	0.04 ± 0.01 b
Oleic acid	C18:1n9c	mg/g	0.14 ± 0.01 a	1.6 ± 0.2 c	1.7 ± 0.1 c	1.2 ± 0.1 b
Linoleic acid	C18:2n6c	mg/g	0.68 ± 0.05 a	2.7 ± 0.3 b	2.6 ± 0.1 b	2.3 ± 0.2 b
α-Linolenic acid	C18:3n3	mg/g	0.03 ± 0.01 a	0.41 ± 0.05 b	0.37 ± 0.02 b	0.36 ± 0.04 b
**Organic Acid or Reference**		**Units ^a^**	**Breads**			
			**Control**	**White Quinoa**	**Red Quinoa**	**Black Quinoa**
Lipids		g/100 g	1.09 ± 0.09 a	2.2 ± 0.1 bc	2.3 ± 0.2 c	2.02 ± 0.07 b
Palmitic acid	C16:0	mg/g	0.29 ± 0.03 a	0.38 ± 0.02 b	0.40 ± 0.03 b	0.36 ± 0.01 b
Stearic acid	C18:0	mg/g	0.02 ± 0.01 a	0.03 ± 0.01 b	0.04 ± 0.01 b	0.03 ± 0.01 b
Oleic acid	C18:1n9c	mg/g	0.14 ± 0.01 a	0.54 ± 0.03 c	0.61 ± 0.06 d	0.46 ± 0.01 b
Linoleic acid	C18:2n6c	mg/g	0.60 ± 0.05 a	1.11 ± 0.06 b	1.18 ± 0.10 b	1.06 ± 0.04 b
α-Linolenic acid	C18:3n3	mg/g	0.09 ± 0.04 a	0.12 ± 0.01 b	0.12 ± 0.02 b	0.11 ± 0.01 b
LA/ALA	C18:2n6c/ C18:3n3	g/g	6.6/1	9.2/1	9.8/1	9.6/1
% of contribution of AI E% for LA	FAO	2.5 E%	8	14	15	14
	EFSA	4.0 E%	5	9	9	9
% of contribution of AI E% for ALA	FAO/EFSA	0.5 E%	6	8	8	7

Values are expressed as mean ± standard deviation (*n* = 3). Values followed by the same letter in the same line are not significantly different at 95% confidence level. ^a^ Dry matter. AI (adequate intake) contribution (%) for a daily average intake of 100 g of bread. AI E% (percentage of energy intake) for LA (linoleic acid) and ALA (α-linolenic acid) for adult ≥ 18, (FAO, 2007; EFSA, 2017), E = (Kcal protein + Kcal lipid + Kcal carbohydrates) in 100 g of bread. EFSA: European Food Safety Authority. FAO: Food and Agriculture Organization.

**Table 3:** Mineral (Fe, Ca, Zn) content, phytate level ratio in raw materials and breads, phytate/mineral molar ratio and average requirement contributions.

Parameter		Units ^a^	Flours			
			Control	White Quinoa	Red Quinoa	Black Quinoa
Ash		g/100 g	0.41 ± 0.19 a	2.37 ± 0.02 b	2.32 ± 0.04 b	2.5 ± 0.03 b
Ca		mg/100 g	20.3 ± 1.6 a	30.4 ± 1.4 c	22.9 ± 1.2 b	33.0 ± 0.8 d
Fe		mg/100 g	0.57 ± 0.07 a	2.5 ± 0.3 b	2.21 ± 0.05 b	2.24 ± 0.07 b
Zn		mg/100 g	0.65 ± 0.11 a	1.8 ± 0.1 b	1.97 ± 0.09 c	1.87 ± 0.07 bc
Ins*P*_6_		mg/100 g	2.9 ± 0.4 a	15.7 ± 1.5 b	15.2 ± 1.0 b	16.9 ± 2.5 b
			**Breads**			
			**Control**	**White Quinoa**	**Red Quinoa**	**Black Quinoa**
Ash		mg/100 g	1.04 ± 0.08 a	1.50 ± 0.01 b	1.51 ± 0.06 b	1.61 ± 0.01 b
Ca		mg/100 g	20.5 ± 1.1 a	21.7 ± 1.9 ab	22.0 ± 1.0 ab	23.5 ± 0.2 b
Fe		mg/100 g	0.69 ± 0.08 a	1.2 ± 0.1 c	1.18 ± 0.03 bc	1.08 ± 0.01 b
Zn		mg/100 g	0.60 ± 0.05 a	1.1 ± 0.1 b	1.09 ± 0.05 b	1.08 ± 0.03 b
Ins*P*_6_		mg/100 g	1.3 ± 0.1 a	3.6 ± 0.3 b	3.71 ± 0.05 b	4.0 ± 0.3 b
Ins*P*_6_/Ca ˂ 0.24		mol/mol	0.06	0.16	0.17	0.17
Ins*P*_6_/Fe ˂ 1.0		mol/mol	1.83	2.86	3.14	3.70
Ins*P*_6_/Zn ˂ 15.0		mol/mol	2.1	3.19	3.40	3.70
**AR contribution**		**mg/day**				
Ca	FAO	1000	3	3	3	3
	EFSA	750	2	2	2	2
Fe	FAO	14	5	9	8	8
	EFSA	11/16	6/4	11/8	11/7	10/7
Zn	FAO_High_	4.2/3	14/20	27/37	26/36	26/36
	FAO_Moderatete_	7.0/4.9	9/12	16/23	16/22	15/22
	FAO_Low_	14.0/9.8	4/6	8/11	8/11	8/11
	EFSA_300_	9.4/7.5	6/8	12/15	12/15	11/14
	EFSA_600_	11.7/9.3	5/6	10/12	9/12	9/12
	EFSA_900_	14/11	4/5	8/10	8/10	8/10
	EFSA_1200_	16.3/12.7	4/5	7/9	7/9	7/8

Values are expressed as mean ± standard deviation (*n* = 3). Values followed by the same letter in the same line are not significantly different at 95% confidence level. ^a^ Dry matter AR (average requirement) contribution (%) for a daily average intake of 100 g of bread. AR in mg per day for males/females ≥18. EFSA: European Food Safety Authority. FAO: Food and Agriculture Organization. The FAO considers three levels of bioavailability of zinc, depending on the phytate (Ins*P*_6_) content in the diet (high, FAO_high_; moderate, FAO_moderate_; and low bioavailability, FAO_low_) [30]. EFSA contemplates four levels of phytate intake per day (300, EFSA_300_; 600, EFSA_600_; 900, EFSA_900_ and 1200 mg per day, EFSA_1200_) [29].

**Table 4:** Dietary fibre content in raw materials and breads and contribution to adequate intake.

Parameter	Units ^a^	Flours			
		Control	White Quinoa	Red Quinoa	Black Quinoa
IDF	g/100 g	3.9 ± 0.7 a	11.26 ± 0.01 b	13.9 ± 0.7 bc	17.4 ± 2.3 c
SDF	g/100 g	1.11 ± 0.01 a	3.37 ± 0.01 c	3.9 ± 0.7 c	2.25 ± 0.01 ab
TDF	g/100 g	5.0 ± 0.7 a	14.63 ± 0.02 b	17.8 ± 1.5 b	19.7 ± 2.3 b
		**Breads**			
		**Control**	**White Quinoa**	**Red Quinoa**	**Black Quinoa**
IDF	g/100 g	4.8 ± 0.7 a	6.38 ± 0.01 b	6.9 ± 0.7 b	9.1 ± 0.7 c
SDF	g/100 g	1.07 ± 0.01 a	2.13 ± 0.01 bc	2.7 ± 0.7 c	1.6 ± 0.7 ab
TDF	g/100 g	5.9 ± 0.7 a	8.51 ± 0.01 b	9.6 ± 1.5 bc	10.66 ± 0.01 bc
SDF:IDF	g/g	1:4.5	1:3.0	1:2.6	1:5.7
AI contribution	%	24	34	38	43

Values are expressed as mean ± standard deviation (*n* = 3). Values followed by the same letter in the same line are not significantly different at 95% confidence level. ^a^ Dry matter. SDF:IDF: 1:2 ratio of soluble/insoluble dietary fibre (Jaime et al., 2002) [25]. AI (adequate intake) contribution (%) for a daily average intake of 100 g of bread. AI in g per day for dietary fibre in adult ≥18 is 25 (EFSA, 2017).

**Table 5:** Effect of quinoa flour addition on glycaemic index and glycaemic load.

Parameter	Units	Breads			
		Control	White Quinoa	Red Quinoa	Black Quinoa
Starch ^a^	%	66.2 ± 1.3 b	61.8 ± 1.7 a	62.6 ± 1.1 a	60.0 ± 2.6 a
AUC		5362 ± 172 c	4578 ± 128 a	4572 ± 28 a	4917 ± 141 b
TSH_90_^b^	%	82 ± 9 a	73 ± 5 a	71 ± 4 a	71 ± 4 a
GI ^b^	%	95 ± 1 c	86 ± 1 a	86.5 ± 0.2 a	90 ± 1 b
pGL ^b^	%	28.0 ± 0.5 d	20.1 ± 0.3 b	23.31 ± 0.08 c	19.4 ± 0.3 a
HC ^c^	SH/min	96 ± 15 a	97 ± 7 a	95 ± 11 a	76 ± 6 a
Slope-LB ^c^	SH/min	0.13 ± 0.05 a	0.35 ± 0.03 b	0.36 ± 0.09 b	0.19 ± 0.03 a

Values are expressed as mean ± standard deviation (*n* = 3). Values followed by the same letter in the same line are not significantly different at 95% confidence level. AUC: area under the curve of starch digestion, TSH_90_: total starch hydrolysed at 90 min, GI: glycaemic index. pGL: predicted glycaemic load, w.b.: wet basis, HC: hydrolysis coefficient, SH: starch hydrolysed, ^a^ Dry basis, ^b^ Wet basis, ^c^ Slope and coefficient of hydrolysis calculated for each sample using Lineweaver–Burk’s transformation of the TSH accumulation curves.

## References

[1] Haros M., Schoenlechner R. (2017). Pseudocereals: Chemistry and Technology.

[2] Ballester-Sánchez J., Gil J.V., Haros C.M., Fernández-Espinar M.T. (2019). Effect of incorporating white, red or black quinoa flours on the total polyphenol content, antioxidant activity and colour of bread. Plant Food Hum. Nutr..

[3] Vega-Galvez A.M., Miranda J., Vergara J., Uribe J., Puente L., Martinez E.A. (2010). Nutrition facts and functional potential of quinoa (Chenopodium quinoa Willd.), an ancient Andean grain: A review. J. Sci. Food Agric..

[4] Repo-Carrasco R., Espinoza C., Jacobsen S.E. (2003). Nutritional value and use of the Andean crops quinoa (Chenopodium quinoa) and kañiwa (Chenopodium pallidicaule). Food Rev. Int..

[5] Lopez H.W., Krespine V., Guy C., Messager A., Demigne C., Remesy C. (2001). Prolonged fermentation of whole wheat sourdough reduces phytate level and increases soluble magnesium. J. Agric. Food Chem..

[6] ADA (2008). Position of the American Dietetic Association: Health Implications of Dietary Fiber. J. Am. Diet. Assoc..

[7] Sanz-Penella J.M., Laparra J.M., Haros M. (2014). Impact of α-amylase during breadmaking on in vitro kinetics of starch hydrolysis and glycaemic index of enriched bread with bran. Plant Foods Hum. Nutr..

[8] Schwingshackl L., Hobl L.P., Hoffmann G. (2015). Effects of low glycaemic index/low glycaemic load vs high glycaemic index/high glycaemic load diets on overweight/obesity and associated risk factors in children and adolescents: A systematic review and meta-analysis. Nutr. J..

[9] Carbajal A., García-Arias M.T., García-Fernández M.C. (2003). Ingestas Recomendadas de Energía y Nutrientes. Nutrición y Dietética.

[10] Aranceta J., Muñoz M., Aranceta J., García-Jalón I. (2004). Objetivos Nutricionales y Guías Dietéticas. Nutrición Aplicada y Dietoterapia.

[11] Ballester-Sánchez J., Yalçın E., Fernández-Espinar M.T., Haros C.M. (2019). Rheological and Thermal Properties of Royal Quinoa and Wheat Flour Blends for Breadmaking. Eur. Food Res. Technol..

[12] AOAC (1996). Method 925.09, 991.43, 996.11, 986.11. Official Methods of Analysis.

[13] ISO/TS (2016). Food Products. Determination of the Total Nitrogen Content by Combustion According to the Dumas Principle and Calculation of the Crude Protein Content. Part 2: Cereals, Pulses and Milled Cereal Products.

[14] AACC (2000). Approved Methods of AACC. Method 08-03, 30-10.

[15] Alaiz M., Navarro J.L., Giron J., Vioque E. (1992). Amino acid analysis by high-performance liquid chromatography after derivatization with diethyl ethoxymethylenemalonate. J. Chromatogr..

[16] Motta C., Castanheira I., Gonzales G.B., Delgado I., Torres D., Santos M., Matos A.S. (2019). Impact of cooking methods and malting on amino acids content in amaranth, buckwheat and quinoa. J. Food. Compos. Anal..

[17] FAO (2007). Protein and Amino Acid Requirements in Human Nutrition.

[18] Garcés R., Mancha M. (1993). One-step lipid extraction and fatty acid methyl esters preparation from fresh plant tissues. Anal. Biochem..

[19] IUPAC—International Union of Pure and Applied Chemistry (1992). Standard Methods for the Analysis of Oils, Fats and Derivatives.

[20] Laparra J.M., Haros M. (2018). Inclusion of whole flour from Latin-American crops into bread formulations as substitute of wheat delays glucose release and uptake. Plant Foods Hum. Nutr..

[21] Wolter A., Hager A.S., Zannini E., Arendt E.K. (2014). Influence of sourdough on in vitro starch digestibility and predicted glycemic indices of gluten-free breads. Food Funct..

[22] Diaz-Valencia Y.K., Alca J.J., Calor-Domingues M.A., Zanabria-Galvez S.J., Cruz S.H.D. (2018). Nutritional composition, total phenolic compounds and antioxidant activity of quinoa (Chenopodium quinoa Willd.) of different colours. Nova Biotechnologica et Chimica.

[23] EU (2017). Codex Circular Letter CL 2017/01/-CPL: Comments at Step 3 on the Proposed Draft Standard for Quinoa. https://ec.europa.eu/food/sites/food/files/safety/docs/codex_cccpl_01_cl_2017-01_quinoa.pdf.

[24] He H.-P., Corke H. (2003). Oil and squalene in Amaranthus grain and leaf. J. Agric. Food Chem..

[25] Jaime L., Mollá E., Fernández A., Martín-Cabreras M.A., López-Andreu F.J., Esteban R.M. (2002). Structural carbohydrate differences and potential source of dietary fiber of onion (*Allium cepa* L.) tissues. J. Agric. Food Chem.

[26] Konishi Y., Arai N., Umeda J., Nishinari K. (2000). Cholesterol Lowering Effect of the Methanol Insoluble Materials from the Quinoa Seed Pericarp. Hydrocolloids.

[27] Salas-Salvado J., Bullo M., Perez-Heras A., Ros E. (2007). Dietary fibre, nuts and cardiovascular diseases. Br. J. Nutr..

[28] FAO (2010). Joint FAO/WHO Food Standards Programme, Secretariat of the CODEX Alimentarius Commission. CODEX Alimentarius (CODEX) Guidelines on Nutrition Labeling CAC/GL 2–1985 as Last Amended 2010.

[29] EFSA (European Food Safety Authority) (2017). Dietary Reference Values for Nutrients: Summary Report.

[30] FAO (2001). Joint FAO/WHO Expert Consultation on Human Vitamin and Mineral Requirements.

[31] De Bruin A. (1964). Investigation of the food value of quinoa and canihua. J. Food Sci..

[32] FAO (2008). Joint FAO/WHO Expert Consultation on Fats and Fatty Acids in Human Nutrition.

[33] Field C.J., Caballero B., Finglas P.M. (2003). Fatty Acids: Dietary Importance. Encyclopedia of Food Science and Nutrition.

[34] Bresson J., Flynn A., Heinonen M., Hulshof K., Korhonen H., Lagiou P., Løvik M., Marchelli R., Martin A., Moseley B. (2009). Review of Labelling Reference Intake Values—Scientific Opinion of the Panel on Dietetic Products, Nutrition and Allergies on a Request from the Commission Related to the Review of Labelling Reference Intake Values for Selected Nutritional Elements. EFSA J..

[35] Mustafa A.F., Seguin P., Gélinas B. (2011). Chemical composition, dietary fibre, tannins and minerals of grain amaranth genotypes. Int. J. Food Sci. Nutr..

[36] Delcour J.A., Hoseney R.C. (2010). Principles of Cereal Science and Technology.

[37] Sanz-Penella J.M., Tamayo-Ramos J.A., Sanz Y., Haros M. (2009). Phytate reduction in bran-enriched bread by phytase-producing bifidobacteria. J. Agric. Food Chem..

[38] Ma G., Jin Y., Plao J., Kok F., Guusie B., Jacobsen E. (2005). Phytate, calcium, iron, and zinc contents and their molar ratios in foods commonly consumed in China. J. Agric. Food Chem..

[39] Li G., Zhu F. (2017). Physicochemical properties of quinoa flour as affected by starch interactions. Food Chem..

[40] Rokka S., Ketoja E., Jarvenpaa E., Tahvonen R. (2013). The glycaemic and C-peptide responses of foods rich in dietary fibre from oat, buckwheat and lingonberry. Int. J. Food Sci. Nutr..

